# 3,3,4,4,5,5-Hexa­fluoro-1,2-bis­[5-(2-fluoro-4′-undecyl­oxybiphenyl-4-yl)-2-methyl-3-thien­yl]cyclo­pentene

**DOI:** 10.1107/S1600536808010635

**Published:** 2008-05-03

**Authors:** Michel Frigoli, Jérôme Marrot, Georg H. Mehl, Chantal Larpent

**Affiliations:** a Université de Versailles Saint-Quentin-en-Yvelines, Institut Lavoisier, UMR CNRS 8180, 45 Avenue des Etats-Unis, 78035 Versailles Cedex, France; b Department of Chemistry, University of Hull, Hull, HU6 7RX, UK

## Abstract

The title compound, C_61_H_68_F_8_O_2_S_2_, is a photochromic liquid crystal based on diaryl­ethene as photoswitchable unit. The F atoms connected to the benzene rings are disordered over two positions; the site-occupation factors refined to 0.830 (3)/0.170 (3). The mol­ecule adopts a photo-active anti­parallel conformation and the distance between the two reactive C atoms of the thio­phene rings is 3.448 (3) Å. The dihedral angles between the central cyclo­pentene ring and the adjacent thio­phene rings are 43.56 (3) and 48.58 (3)°. These structural elements exhibit a suitable geometry for photochromic behaviour in the crystalline state.

## Related literature

For general background, see: Irie (2000 ▶); Tian & Yang (2004 ▶); Tian & Wang (2007 ▶); Frigoli *et al.* (2004 ▶). For related literature, see: Morimoto & Irie (2005 ▶); Kobatake *et al.* (2007 ▶).
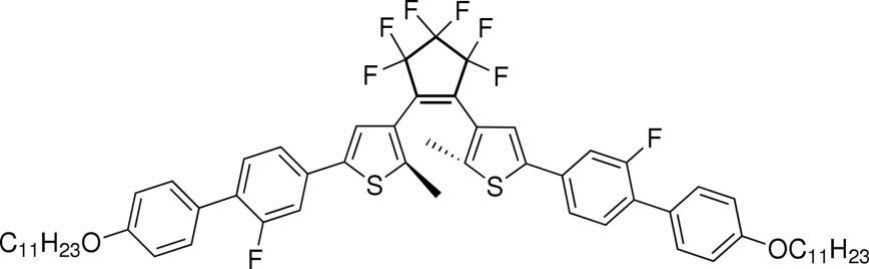


## Experimental

### 

#### Crystal data

C_61_H_68_F_8_O_2_S_2_
*M* *_r_* = 1049.27
Triclinic, 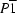

*a* = 10.2324 (15) Å
*b* = 10.9626 (16) Å
*c* = 24.468 (4) Å
α = 77.083 (7)°
β = 85.576 (10)°
γ = 89.924 (7)°
*V* = 2666.9 (7) Å^3^
*Z* = 2
Mo *K*α radiation
μ = 0.17 mm^−1^
*T* = 100 (2) K
0.30 × 0.10 × 0.06 mm

#### Data collection

Bruker Kappa APEXII CCD diffractometer
Absorption correction: multi-scan (*SADABS*; Bruker, 2004 ▶) *T*_min_ = 0.950, *T*_max_ = 0.990
66708 measured reflections
15658 independent reflections
13169 reflections with *I* > 2σ(*I*)
*R*_int_ = 0.030

#### Refinement

*R*[*F* ^2^ > 2σ(*F* ^2^)] = 0.049
*wR*(*F* ^2^) = 0.156
*S* = 1.05
15658 reflections
682 parameters
2 restraints
H-atom parameters constrained
Δρ_max_ = 2.07 e Å^−3^
Δρ_min_ = −0.76 e Å^−3^

### 

Data collection: *APEX2* (Bruker, 2004 ▶); cell refinement: *APEX2*; data reduction: *SAINT* (Bruker, 2004 ▶); program(s) used to solve structure: *SHELXS97* (Sheldrick, 2008 ▶); program(s) used to refine structure: *SHELXL97* (Sheldrick, 2008 ▶); molecular graphics: *SHELXTL* (Sheldrick, 2008 ▶; software used to prepare material for publication: *SHELXTL*.

## Supplementary Material

Crystal structure: contains datablocks I, global. DOI: 10.1107/S1600536808010635/hk2453sup1.cif

Structure factors: contains datablocks I. DOI: 10.1107/S1600536808010635/hk2453Isup2.hkl

Additional supplementary materials:  crystallographic information; 3D view; checkCIF report

**Fig. 2.**
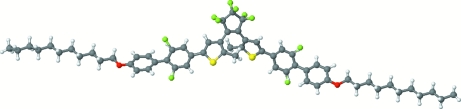
Supplementary figure.

Enhanced figure: interactive version of Fig. 2

## Figures and Tables

**Fig. 1.**
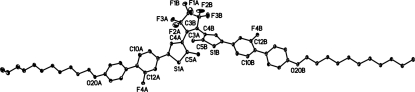
The molecular structure of the title molecule, with the atom-numbering scheme only for the most relevant atoms. Displacement ellipsoids are drawn at the 50% probability level. Only the major component is shown, for clarity.

## supplementary crystallographic information

### Comment

Diarylethene molecules are known to be the most promising organic photochromic material for three-dimensional optical memory and molecular switches (Irie, 2000; Tian & Yang, 2004; Tian & Wang, 2007). The title compound, (I), was the first photochromic liquid crystal based on diarylethene material (Frigoli *et al.*,  2004). We have shown that photochromic behaviour occurs very efficiently in solution and in the liquid crystalline state.

The molecule of the title compound, (I), (Fig. 1) adopts an antiparallel conformation. When the crystal structure was solved, the F atoms connected to the phenyl groups were found to be disordered.

The C5A···C5B [3.448 (2) Å] distance between the carbons bearing the methyl groups of the thiophene rings indicates that the photochromic reaction can occur in the crystalline state as well (Morimoto & Irie, 2005; Kobatake *et al.*,  2007). The dihedral angles between the central cyclopentene ring: A (C1A-C3A/C2B/C3B) and the adjacent thiophene rings: B (S1A/C4A-C7A) and C (S1B/C4B-C7B) are 43.56 (3)° and 48.58 (3)°, respectively.

### Experimental

The synthesis of (I) was reported, previously (Frigoli *et al.*, 2004). The needle-like crystals of (I) suitable for X-ray analysis were obtained by slow evaporation of a cyclohexane solution.

### Refinement

When the crystal structure was solved, the F atoms connected to the phenyl groups were found to be disordered. They were each modelled with disorder over two positions and their occupancies were refined as 0.830 (3) (for F4A), 0.170 (3) (for F5A), 0.790 (3) (for F4B) and 0.210 (3) (for F5B). The C-F bonds between the F atoms and the phenyl groups with the lowest site occupancy were restrained to be 1.34 Å. H atoms were positioned geometrically, with C-H = 0.95, 0.99 and 0.98 Å for aromatic, methylene and methyl H, respectively, and constrained to ride on their parent atoms with U_iso_(H) = xU_eq_(C), where x = 1.5 for methyl H and x = 1.2 for all other H atoms.

### Crystal data

**Table:**

C_61_H_68_F_8_O_2_S_2_	*Z* = 2
*M**_r_* = 1049.27	*F*_000_ = 1108
Triclinic, *P*1	*D*_x_ = 1.307 Mg m^−^^3^
Hall symbol: -P 1	Melting point: 372.8 K
*a* = 10.2324 (15) Å	Mo *K*α radiation λ = 0.71073 Å
*b* = 10.9626 (16) Å	Cell parameters from 9287 reflections
*c* = 24.468 (4) Å	θ = 2.5–30.1º
α = 77.083 (7)º	µ = 0.17 mm^−^^1^
β = 85.576 (10)º	*T* = 100 (2) K
γ = 89.924 (7)º	Needle, light blue
*V* = 2666.9 (7) Å^3^	0.30 × 0.10 × 0.06 mm

### Data collection

**Table:**

Bruker Kappa APEXII CCD diffractometer	15658 independent reflections
Radiation source: fine-focus sealed tube	13169 reflections with *I* > 2σ(*I*)
Monochromator: graphite	*R*_int_ = 0.030
Detector resolution: 512x512 pixels mm^-1^	θ_max_ = 30.1º
*T* = 100(2) K	θ_min_ = 0.9º
φ and ω scans	*h* = −14→14
Absorption correction: multi-scan(SADABS; Bruker, 2004)	*k* = −15→15
*T*_min_ = 0.950, *T*_max_ = 0.990	*l* = −34→34
66708 measured reflections	

### Refinement

**Table:**

Refinement on *F*^2^	Secondary atom site location: difference Fourier map
Least-squares matrix: full	Hydrogen site location: inferred from neighbouring sites
*R*[*F*^2^ > 2σ(*F*^2^)] = 0.049	H-atom parameters constrained
*wR*(*F*^2^) = 0.156	*w* = 1/[σ^2^(*F*_o_^2^) + (0.0897*P*)^2^ + 1.5953*P*] where *P* = (*F*_o_^2^ + 2*F*_c_^2^)/3
*S* = 1.05	(Δ/σ)_max_ = 0.001
15658 reflections	Δρ_max_ = 2.07 e Å^−^^3^
682 parameters	Δρ_min_ = −0.76 e Å^−^^3^
2 restraints	Extinction correction: none
Primary atom site location: structure-invariant direct methods	

### Special details

**Table:**

Geometry. All e.s.d.'s (except the e.s.d. in the dihedral angle between two l.s. planes) are estimated using the full covariance matrix. The cell e.s.d.'s are taken into account individually in the estimation of e.s.d.'s in distances, angles and torsion angles; correlations between e.s.d.'s in cell parameters are only used when they are defined by crystal symmetry. An approximate (isotropic) treatment of cell e.s.d.'s is used for estimating e.s.d.'s involving l.s. planes.
Refinement. Refinement of F^2^ against ALL reflections. The weighted R-factor wR and goodness of fit S are based on F^2^, conventional R-factors R are based on F, with F set to zero for negative F^2^. The threshold expression of F^2^ > 2sigma(F^2^) is used only for calculating R-factors(gt) etc. and is not relevant to the choice of reflections for refinement. R-factors based on F^2^ are statistically about twice as large as those based on F, and R- factors based on ALL data will be even larger.

### Fractional atomic coordinates and isotropic or equivalent isotropic displacement parameters (Å^2^)

**Table:**

	*x*	*y*	*z*	*U*_iso_*/*U*_eq_	Occ. (<1)
S1A	0.26666 (4)	0.32993 (3)	0.130497 (14)	0.01950 (9)	
S1B	0.36216 (4)	0.54474 (3)	−0.097570 (15)	0.01946 (9)	
F1A	0.09780 (15)	−0.07874 (14)	−0.02856 (6)	0.0572 (4)	
F2A	0.17004 (15)	−0.06215 (11)	0.06498 (5)	0.0484 (3)	
F3A	0.37487 (13)	−0.06276 (10)	0.03551 (5)	0.0424 (3)	
F1B	0.29892 (17)	−0.12047 (13)	−0.05048 (6)	0.0576 (4)	
F2B	0.33540 (19)	0.09210 (13)	−0.11450 (6)	0.0696 (5)	
F3B	0.12612 (17)	0.11540 (11)	−0.10413 (6)	0.0586 (4)	
C1A	0.22190 (18)	−0.03564 (15)	−0.03392 (7)	0.0274 (3)	
C2A	0.25788 (17)	−0.00932 (14)	0.02232 (7)	0.0245 (3)	
C3A	0.26556 (14)	0.13067 (13)	0.01336 (6)	0.0180 (3)	
C4A	0.28225 (14)	0.18778 (13)	0.06092 (6)	0.0179 (3)	
C5A	0.21719 (14)	0.29210 (14)	0.07076 (6)	0.0189 (3)	
C6A	0.36934 (14)	0.13842 (13)	0.10326 (6)	0.0185 (3)	
H6A	0.4202	0.0660	0.1030	0.022*	
C7A	0.37236 (14)	0.20524 (13)	0.14403 (6)	0.0176 (3)	
C8A	0.44329 (14)	0.17614 (14)	0.19500 (6)	0.0174 (3)	
C9A	0.52927 (16)	0.07574 (17)	0.20384 (6)	0.0257 (3)	
H9A	0.5461	0.0300	0.1754	0.031*	
C10A	0.59008 (15)	0.04217 (16)	0.25338 (6)	0.0237 (3)	
H10A	0.6466	−0.0276	0.2582	0.028*	0.830 (3)
F5A	0.6357 (6)	−0.0878 (5)	0.2634 (2)	0.0272 (16)	0.170 (3)
C11A	0.57218 (14)	0.10607 (13)	0.29668 (6)	0.0173 (3)	
C12A	0.48413 (16)	0.20477 (13)	0.28672 (6)	0.0204 (3)	
H12A	0.4664	0.2498	0.3154	0.024*	0.170 (3)
F4A	0.45068 (12)	0.26923 (11)	0.32778 (5)	0.0257 (3)	0.830 (3)
C13A	0.42129 (16)	0.24093 (13)	0.23775 (6)	0.0206 (3)	
H13A	0.3634	0.3096	0.2333	0.025*	
C14A	0.64219 (14)	0.06645 (13)	0.34849 (6)	0.0165 (2)	
C15A	0.76122 (14)	0.00391 (14)	0.34597 (6)	0.0182 (3)	
H15A	0.7986	−0.0056	0.3104	0.022*	
C16A	0.82662 (14)	−0.04480 (13)	0.39369 (6)	0.0170 (3)	
H16A	0.9063	−0.0882	0.3907	0.020*	
C17A	0.77412 (13)	−0.02928 (12)	0.44576 (6)	0.0155 (2)	
C18A	0.65849 (14)	0.03770 (13)	0.44916 (6)	0.0171 (3)	
H18A	0.6242	0.0512	0.4845	0.021*	
C19A	0.59345 (14)	0.08458 (13)	0.40136 (6)	0.0172 (3)	
H19A	0.5148	0.1296	0.4044	0.021*	
O20A	0.82961 (10)	−0.07467 (10)	0.49529 (4)	0.0181 (2)	
C21A	0.94176 (14)	−0.15368 (13)	0.49318 (6)	0.0168 (2)	
H21A	1.0154	−0.1060	0.4692	0.020*	
H21B	0.9195	−0.2258	0.4772	0.020*	
C22A	0.97981 (14)	−0.19882 (13)	0.55275 (6)	0.0167 (2)	
H22A	0.9043	−0.2444	0.5762	0.020*	
H22B	1.0000	−0.1254	0.5681	0.020*	
C23A	1.09815 (14)	−0.28464 (13)	0.55689 (6)	0.0176 (3)	
H23A	1.0773	−0.3598	0.5429	0.021*	
H23B	1.1733	−0.2403	0.5327	0.021*	
C24A	1.13636 (14)	−0.32495 (13)	0.61733 (6)	0.0170 (2)	
H24A	1.0599	−0.3674	0.6413	0.020*	
H24B	1.1571	−0.2492	0.6309	0.020*	
C25A	1.25329 (14)	−0.41233 (14)	0.62483 (6)	0.0187 (3)	
H25A	1.2339	−0.4880	0.6109	0.022*	
H25B	1.3310	−0.3696	0.6020	0.022*	
C26A	1.28462 (14)	−0.45134 (14)	0.68623 (6)	0.0190 (3)	
H26A	1.2073	−0.4959	0.7086	0.023*	
H26B	1.3001	−0.3750	0.7003	0.023*	
C27A	1.40379 (16)	−0.53541 (16)	0.69600 (6)	0.0236 (3)	
H27A	1.3857	−0.6149	0.6850	0.028*	
H27B	1.4799	−0.4939	0.6715	0.028*	
C28A	1.43891 (15)	−0.56448 (16)	0.75704 (6)	0.0241 (3)	
H28A	1.4302	−0.4871	0.7715	0.029*	
H28B	1.3748	−0.6268	0.7797	0.029*	
C29A	1.57607 (19)	−0.6145 (2)	0.76576 (8)	0.0346 (4)	
H29A	1.6405	−0.5528	0.7429	0.041*	
H29B	1.5848	−0.6926	0.7520	0.041*	
C30A	1.6090 (2)	−0.6412 (2)	0.82714 (8)	0.0358 (4)	
H30A	1.5893	−0.5660	0.8422	0.043*	
H30B	1.5512	−0.7102	0.8490	0.043*	
C31A	1.7497 (2)	−0.6764 (2)	0.83619 (9)	0.0418 (5)	
H31A	1.7675	−0.7562	0.8256	0.063*	
H31B	1.7647	−0.6846	0.8759	0.063*	
H31C	1.8082	−0.6111	0.8130	0.063*	
C32A	0.11312 (16)	0.36790 (16)	0.03948 (6)	0.0242 (3)	
H32A	0.1541	0.4384	0.0116	0.036*	
H32B	0.0530	0.3997	0.0660	0.036*	
H32C	0.0642	0.3151	0.0204	0.036*	
C2B	0.23353 (19)	0.09292 (15)	−0.07575 (7)	0.0270 (3)	
C3B	0.25741 (14)	0.18662 (13)	−0.04149 (6)	0.0177 (3)	
C4B	0.27481 (14)	0.31989 (13)	−0.06856 (5)	0.0166 (3)	
C5B	0.37792 (14)	0.39256 (13)	−0.06085 (6)	0.0175 (3)	
C6B	0.18441 (14)	0.38799 (13)	−0.10536 (6)	0.0172 (3)	
H6B	0.1092	0.3508	−0.1158	0.021*	
C7B	0.21740 (14)	0.51230 (13)	−0.12398 (6)	0.0172 (3)	
C8B	0.14597 (14)	0.61043 (13)	−0.16030 (6)	0.0172 (3)	
C9B	0.16133 (15)	0.73655 (14)	−0.15960 (6)	0.0221 (3)	
H9B	0.2222	0.7607	−0.1364	0.026*	
C10B	0.08821 (15)	0.82686 (14)	−0.19256 (7)	0.0215 (3)	
H10B	0.1002	0.9120	−0.1912	0.026*	0.790 (3)
F5B	0.1051 (5)	0.9398 (4)	−0.1872 (2)	0.0331 (15)	0.210 (3)
C11B	−0.00246 (14)	0.79714 (13)	−0.22761 (6)	0.0165 (2)	
C12B	−0.01302 (14)	0.67084 (13)	−0.22799 (6)	0.0167 (3)	
H12B	−0.0723	0.6469	−0.2519	0.020*	0.210 (3)
F4B	−0.10195 (11)	0.63417 (10)	−0.25907 (5)	0.0214 (3)	0.790 (3)
C13B	0.05760 (14)	0.57843 (13)	−0.19565 (6)	0.0174 (3)	
H13B	0.0461	0.4935	−0.1974	0.021*	
C14B	−0.08765 (13)	0.89257 (13)	−0.25938 (6)	0.0161 (2)	
C15B	−0.15027 (14)	0.98035 (13)	−0.23334 (6)	0.0176 (3)	
H15B	−0.1345	0.9813	−0.1957	0.021*	
C16B	−0.23517 (14)	1.06622 (13)	−0.26184 (6)	0.0185 (3)	
H16B	−0.2782	1.1244	−0.2434	0.022*	
C17B	−0.25764 (14)	1.06759 (13)	−0.31750 (6)	0.0169 (3)	
C18B	−0.19397 (14)	0.98259 (13)	−0.34459 (6)	0.0182 (3)	
H18B	−0.2069	0.9841	−0.3828	0.022*	
C19B	−0.11097 (14)	0.89513 (13)	−0.31500 (6)	0.0183 (3)	
H19B	−0.0693	0.8358	−0.3332	0.022*	
O20B	−0.34398 (11)	1.15515 (10)	−0.34106 (4)	0.0201 (2)	
C21B	−0.36989 (14)	1.16370 (13)	−0.39837 (6)	0.0181 (3)	
H21C	−0.3933	1.0802	−0.4042	0.022*	
H21D	−0.2915	1.1962	−0.4237	0.022*	
C22B	−0.48311 (14)	1.25237 (13)	−0.41046 (6)	0.0182 (3)	
H22C	−0.4615	1.3320	−0.4003	0.022*	
H22D	−0.5619	1.2155	−0.3864	0.022*	
C23B	−0.51473 (14)	1.27997 (13)	−0.47178 (6)	0.0177 (3)	
H23C	−0.5354	1.2004	−0.4823	0.021*	
H23D	−0.4367	1.3184	−0.4959	0.021*	
C24B	−0.63045 (14)	1.36794 (13)	−0.48255 (6)	0.0175 (3)	
H24C	−0.7083	1.3286	−0.4586	0.021*	
H24D	−0.6100	1.4465	−0.4711	0.021*	
C25B	−0.66411 (14)	1.39987 (13)	−0.54375 (6)	0.0184 (3)	
H25C	−0.6875	1.3217	−0.5549	0.022*	
H25D	−0.5857	1.4371	−0.5679	0.022*	
C26B	−0.77763 (14)	1.49103 (13)	−0.55398 (6)	0.0180 (3)	
H26C	−0.8571	1.4519	−0.5312	0.022*	
H26D	−0.7560	1.5673	−0.5410	0.022*	
C27B	−0.80802 (14)	1.52863 (13)	−0.61557 (6)	0.0186 (3)	
H27C	−0.8288	1.4522	−0.6286	0.022*	
H27D	−0.7287	1.5683	−0.6383	0.022*	
C28B	−0.92223 (14)	1.61898 (13)	−0.62599 (6)	0.0182 (3)	
H28C	−1.0007	1.5813	−0.6019	0.022*	
H28D	−0.8997	1.6975	−0.6149	0.022*	
C29B	−0.95494 (15)	1.64984 (13)	−0.68731 (6)	0.0185 (3)	
H29C	−0.9823	1.5718	−0.6975	0.022*	
H29D	−0.8748	1.6823	−0.7114	0.022*	
C30B	−1.06341 (15)	1.74599 (14)	−0.69963 (6)	0.0206 (3)	
H30C	−1.1428	1.7152	−0.6746	0.025*	
H30D	−1.0348	1.8254	−0.6910	0.025*	
C31B	−1.09804 (17)	1.77157 (15)	−0.76061 (7)	0.0256 (3)	
H31D	−1.0197	1.8015	−0.7856	0.038*	
H31E	−1.1658	1.8354	−0.7665	0.038*	
H31F	−1.1309	1.6942	−0.7689	0.038*	
C32B	0.49542 (15)	0.35625 (14)	−0.02802 (6)	0.0215 (3)	
H32D	0.4769	0.3679	0.0104	0.032*	
H32E	0.5708	0.4089	−0.0462	0.032*	
H32F	0.5153	0.2682	−0.0268	0.032*	

### Atomic displacement parameters (Å^2^)

**Table:**

	*U*^11^	*U*^22^	*U*^33^	*U*^12^	*U*^13^	*U*^23^
S1A	0.02037 (17)	0.02434 (17)	0.01362 (15)	0.00758 (13)	−0.00211 (12)	−0.00369 (12)
S1B	0.01891 (17)	0.01874 (16)	0.01937 (16)	0.00367 (12)	−0.00647 (12)	0.00022 (12)
F1A	0.0544 (8)	0.0580 (8)	0.0514 (8)	−0.0252 (7)	−0.0263 (7)	0.0115 (6)
F2A	0.0741 (9)	0.0307 (6)	0.0353 (6)	−0.0165 (6)	0.0131 (6)	−0.0021 (5)
F3A	0.0556 (7)	0.0267 (5)	0.0529 (7)	0.0245 (5)	−0.0357 (6)	−0.0153 (5)
F1B	0.0931 (11)	0.0451 (7)	0.0451 (7)	0.0445 (8)	−0.0282 (7)	−0.0250 (6)
F2B	0.1148 (14)	0.0413 (7)	0.0533 (8)	−0.0194 (8)	0.0472 (9)	−0.0302 (6)
F3B	0.0945 (11)	0.0276 (6)	0.0606 (8)	0.0042 (6)	−0.0611 (8)	−0.0060 (5)
C1A	0.0342 (9)	0.0187 (7)	0.0315 (8)	0.0055 (6)	−0.0124 (7)	−0.0070 (6)
C2A	0.0314 (8)	0.0183 (6)	0.0227 (7)	0.0048 (6)	−0.0072 (6)	−0.0006 (5)
C3A	0.0196 (6)	0.0172 (6)	0.0168 (6)	0.0059 (5)	−0.0044 (5)	−0.0020 (5)
C4A	0.0197 (6)	0.0197 (6)	0.0124 (6)	0.0046 (5)	−0.0030 (5)	0.0006 (5)
C5A	0.0188 (6)	0.0237 (7)	0.0127 (6)	0.0059 (5)	−0.0021 (5)	−0.0009 (5)
C6A	0.0201 (6)	0.0196 (6)	0.0144 (6)	0.0052 (5)	−0.0041 (5)	0.0003 (5)
C7A	0.0159 (6)	0.0208 (6)	0.0139 (6)	0.0033 (5)	−0.0017 (5)	0.0008 (5)
C8A	0.0146 (6)	0.0217 (6)	0.0143 (6)	0.0007 (5)	−0.0019 (5)	−0.0001 (5)
C9A	0.0257 (8)	0.0368 (8)	0.0166 (6)	0.0147 (6)	−0.0060 (6)	−0.0091 (6)
C10A	0.0217 (7)	0.0325 (8)	0.0180 (6)	0.0135 (6)	−0.0058 (5)	−0.0062 (6)
F5A	0.034 (3)	0.023 (3)	0.030 (3)	0.018 (2)	−0.019 (2)	−0.011 (2)
C11A	0.0148 (6)	0.0200 (6)	0.0156 (6)	−0.0008 (5)	−0.0034 (5)	−0.0002 (5)
C12A	0.0268 (7)	0.0165 (6)	0.0192 (6)	0.0022 (5)	−0.0075 (5)	−0.0048 (5)
F4A	0.0337 (7)	0.0251 (6)	0.0229 (6)	0.0124 (5)	−0.0112 (5)	−0.0126 (4)
C13A	0.0258 (7)	0.0164 (6)	0.0200 (6)	0.0033 (5)	−0.0088 (5)	−0.0024 (5)
C14A	0.0154 (6)	0.0165 (6)	0.0167 (6)	−0.0012 (5)	−0.0044 (5)	−0.0005 (5)
C15A	0.0156 (6)	0.0236 (6)	0.0146 (6)	0.0011 (5)	−0.0024 (5)	−0.0021 (5)
C16A	0.0148 (6)	0.0201 (6)	0.0156 (6)	0.0022 (5)	−0.0030 (5)	−0.0020 (5)
C17A	0.0151 (6)	0.0156 (6)	0.0150 (6)	−0.0005 (5)	−0.0046 (5)	−0.0007 (4)
C18A	0.0160 (6)	0.0180 (6)	0.0169 (6)	0.0005 (5)	−0.0017 (5)	−0.0028 (5)
C19A	0.0147 (6)	0.0171 (6)	0.0192 (6)	0.0007 (5)	−0.0035 (5)	−0.0019 (5)
O20A	0.0182 (5)	0.0216 (5)	0.0140 (4)	0.0049 (4)	−0.0047 (4)	−0.0016 (4)
C21A	0.0167 (6)	0.0174 (6)	0.0160 (6)	0.0027 (5)	−0.0041 (5)	−0.0020 (5)
C22A	0.0169 (6)	0.0172 (6)	0.0151 (6)	0.0009 (5)	−0.0040 (5)	−0.0006 (5)
C23A	0.0179 (6)	0.0179 (6)	0.0166 (6)	0.0017 (5)	−0.0040 (5)	−0.0023 (5)
C24A	0.0160 (6)	0.0187 (6)	0.0159 (6)	0.0025 (5)	−0.0037 (5)	−0.0024 (5)
C25A	0.0178 (6)	0.0205 (6)	0.0176 (6)	0.0043 (5)	−0.0027 (5)	−0.0033 (5)
C26A	0.0173 (6)	0.0216 (6)	0.0175 (6)	0.0047 (5)	−0.0028 (5)	−0.0030 (5)
C27A	0.0217 (7)	0.0314 (8)	0.0176 (6)	0.0118 (6)	−0.0048 (5)	−0.0042 (6)
C28A	0.0187 (7)	0.0342 (8)	0.0184 (6)	0.0087 (6)	−0.0040 (5)	−0.0026 (6)
C29A	0.0329 (9)	0.0446 (10)	0.0266 (8)	0.0156 (8)	−0.0069 (7)	−0.0072 (7)
C30A	0.0392 (10)	0.0419 (10)	0.0244 (8)	0.0086 (8)	−0.0034 (7)	−0.0032 (7)
C31A	0.0403 (11)	0.0533 (12)	0.0333 (10)	0.0103 (9)	−0.0107 (8)	−0.0103 (9)
C32A	0.0245 (7)	0.0315 (8)	0.0166 (6)	0.0146 (6)	−0.0045 (5)	−0.0046 (6)
C2B	0.0416 (9)	0.0225 (7)	0.0182 (7)	0.0036 (6)	−0.0067 (6)	−0.0059 (5)
C3B	0.0199 (6)	0.0182 (6)	0.0154 (6)	0.0059 (5)	−0.0039 (5)	−0.0036 (5)
C4B	0.0198 (6)	0.0180 (6)	0.0118 (5)	0.0072 (5)	−0.0032 (5)	−0.0025 (4)
C5B	0.0196 (6)	0.0186 (6)	0.0139 (6)	0.0067 (5)	−0.0031 (5)	−0.0019 (5)
C6B	0.0184 (6)	0.0199 (6)	0.0131 (5)	0.0053 (5)	−0.0045 (5)	−0.0021 (5)
C7B	0.0174 (6)	0.0197 (6)	0.0139 (6)	0.0053 (5)	−0.0042 (5)	−0.0017 (5)
C8B	0.0168 (6)	0.0194 (6)	0.0135 (6)	0.0055 (5)	−0.0027 (5)	0.0007 (5)
C9B	0.0220 (7)	0.0207 (6)	0.0228 (7)	0.0021 (5)	−0.0109 (5)	−0.0001 (5)
C10B	0.0214 (7)	0.0171 (6)	0.0245 (7)	0.0017 (5)	−0.0083 (5)	0.0007 (5)
F5B	0.035 (3)	0.013 (2)	0.052 (3)	0.0023 (17)	−0.022 (2)	−0.0014 (19)
C11B	0.0154 (6)	0.0173 (6)	0.0147 (6)	0.0029 (5)	−0.0020 (5)	0.0015 (5)
C12B	0.0162 (6)	0.0201 (6)	0.0136 (5)	0.0049 (5)	−0.0037 (5)	−0.0027 (5)
F4B	0.0238 (6)	0.0208 (6)	0.0222 (6)	0.0055 (4)	−0.0122 (4)	−0.0069 (4)
C13B	0.0185 (6)	0.0176 (6)	0.0157 (6)	0.0057 (5)	−0.0031 (5)	−0.0025 (5)
C14B	0.0143 (6)	0.0162 (6)	0.0155 (6)	0.0018 (5)	−0.0026 (5)	0.0018 (5)
C15B	0.0193 (6)	0.0158 (6)	0.0167 (6)	0.0010 (5)	−0.0049 (5)	−0.0003 (5)
C16B	0.0202 (6)	0.0142 (6)	0.0209 (6)	0.0030 (5)	−0.0048 (5)	−0.0022 (5)
C17B	0.0149 (6)	0.0143 (6)	0.0195 (6)	0.0014 (5)	−0.0050 (5)	0.0016 (5)
C18B	0.0165 (6)	0.0201 (6)	0.0157 (6)	0.0033 (5)	−0.0024 (5)	0.0011 (5)
C19B	0.0168 (6)	0.0204 (6)	0.0156 (6)	0.0048 (5)	−0.0008 (5)	0.0004 (5)
O20B	0.0224 (5)	0.0175 (5)	0.0195 (5)	0.0068 (4)	−0.0081 (4)	−0.0003 (4)
C21B	0.0175 (6)	0.0166 (6)	0.0194 (6)	0.0020 (5)	−0.0071 (5)	−0.0005 (5)
C22B	0.0165 (6)	0.0167 (6)	0.0200 (6)	0.0032 (5)	−0.0064 (5)	0.0007 (5)
C23B	0.0166 (6)	0.0153 (6)	0.0206 (6)	0.0028 (5)	−0.0065 (5)	−0.0011 (5)
C24B	0.0158 (6)	0.0162 (6)	0.0198 (6)	0.0029 (5)	−0.0063 (5)	−0.0008 (5)
C25B	0.0170 (6)	0.0174 (6)	0.0204 (6)	0.0029 (5)	−0.0068 (5)	−0.0014 (5)
C26B	0.0165 (6)	0.0171 (6)	0.0195 (6)	0.0018 (5)	−0.0061 (5)	−0.0005 (5)
C27B	0.0177 (6)	0.0176 (6)	0.0199 (6)	0.0029 (5)	−0.0073 (5)	−0.0010 (5)
C28B	0.0179 (6)	0.0181 (6)	0.0181 (6)	0.0029 (5)	−0.0065 (5)	−0.0014 (5)
C29B	0.0210 (7)	0.0153 (6)	0.0194 (6)	0.0030 (5)	−0.0077 (5)	−0.0019 (5)
C30B	0.0219 (7)	0.0192 (6)	0.0204 (6)	0.0047 (5)	−0.0082 (5)	−0.0016 (5)
C31B	0.0314 (8)	0.0222 (7)	0.0237 (7)	0.0061 (6)	−0.0138 (6)	−0.0022 (6)
C32B	0.0212 (7)	0.0218 (7)	0.0214 (7)	0.0068 (5)	−0.0080 (5)	−0.0028 (5)

### Geometric parameters (Å, °)

**Table:**

S1A—C5A	1.7176 (15)	C31A—H31A	0.9800
S1A—C7A	1.7311 (14)	C31A—H31B	0.9800
S1B—C5B	1.7228 (14)	C31A—H31C	0.9800
S1B—C7B	1.7292 (15)	C32A—H32A	0.9800
F1A—C1A	1.342 (2)	C32A—H32B	0.9800
F2A—C2A	1.349 (2)	C32A—H32C	0.9800
F3A—C2A	1.3616 (19)	C2B—C3B	1.495 (2)
F1B—C1A	1.329 (2)	C3B—C4B	1.4680 (19)
F2B—C2B	1.355 (2)	C4B—C5B	1.372 (2)
F3B—C2B	1.339 (2)	C4B—C6B	1.4316 (18)
C1A—C2A	1.539 (2)	C5B—C32B	1.5014 (19)
C1A—C2B	1.544 (2)	C6B—C7B	1.371 (2)
C2A—C3A	1.502 (2)	C6B—H6B	0.9500
C3A—C3B	1.3540 (19)	C7B—C8B	1.4669 (18)
C3A—C4A	1.460 (2)	C8B—C13B	1.395 (2)
C4A—C5A	1.380 (2)	C8B—C9B	1.396 (2)
C4A—C6A	1.4320 (18)	C9B—C10B	1.3874 (19)
C5A—C32A	1.4999 (19)	C9B—H9B	0.9500
C6A—C7A	1.365 (2)	C10B—F5B	1.287 (5)
C6A—H6A	0.9500	C10B—C11B	1.397 (2)
C7A—C8A	1.4645 (19)	C10B—H10B	0.9500
C8A—C13A	1.395 (2)	C11B—C12B	1.391 (2)
C8A—C9A	1.397 (2)	C11B—C14B	1.4822 (18)
C9A—C10A	1.381 (2)	C12B—F4B	1.3463 (18)
C9A—H9A	0.9500	C12B—C13B	1.3793 (18)
C10A—C11A	1.396 (2)	C12B—H12B	0.9500
C10A—F5A	1.473 (5)	C13B—H13B	0.9500
C10A—H10A	0.9500	C14B—C19B	1.3939 (19)
C11A—C12A	1.398 (2)	C14B—C15B	1.397 (2)
C11A—C14A	1.4840 (18)	C15B—C16B	1.3874 (18)
C12A—F4A	1.3745 (18)	C15B—H15B	0.9500
C12A—C13A	1.383 (2)	C16B—C17B	1.395 (2)
C12A—H12A	0.9500	C16B—H16B	0.9500
C13A—H13A	0.9500	C17B—O20B	1.3649 (16)
C14A—C15A	1.401 (2)	C17B—C18B	1.393 (2)
C14A—C19A	1.406 (2)	C18B—C19B	1.3953 (18)
C15A—C16A	1.3918 (18)	C18B—H18B	0.9500
C15A—H15A	0.9500	C19B—H19B	0.9500
C16A—C17A	1.3898 (19)	O20B—C21B	1.4295 (17)
C16A—H16A	0.9500	C21B—C22B	1.5152 (19)
C17A—O20A	1.3691 (16)	C21B—H21C	0.9900
C17A—C18A	1.3985 (19)	C21B—H21D	0.9900
C18A—C19A	1.3876 (19)	C22B—C23B	1.523 (2)
C18A—H18A	0.9500	C22B—H22C	0.9900
C19A—H19A	0.9500	C22B—H22D	0.9900
O20A—C21A	1.4415 (17)	C23B—C24B	1.5274 (19)
C21A—C22A	1.5118 (18)	C23B—H23C	0.9900
C21A—H21A	0.9900	C23B—H23D	0.9900
C21A—H21B	0.9900	C24B—C25B	1.5260 (19)
C22A—C23A	1.5281 (19)	C24B—H24C	0.9900
C22A—H22A	0.9900	C24B—H24D	0.9900
C22A—H22B	0.9900	C25B—C26B	1.5299 (19)
C23A—C24A	1.5270 (19)	C25B—H25C	0.9900
C23A—H23A	0.9900	C25B—H25D	0.9900
C23A—H23B	0.9900	C26B—C27B	1.5269 (19)
C24A—C25A	1.5281 (19)	C26B—H26C	0.9900
C24A—H24A	0.9900	C26B—H26D	0.9900
C24A—H24B	0.9900	C27B—C28B	1.5303 (19)
C25A—C26A	1.526 (2)	C27B—H27C	0.9900
C25A—H25A	0.9900	C27B—H27D	0.9900
C25A—H25B	0.9900	C28B—C29B	1.5256 (19)
C26A—C27A	1.529 (2)	C28B—H28C	0.9900
C26A—H26A	0.9900	C28B—H28D	0.9900
C26A—H26B	0.9900	C29B—C30B	1.5297 (19)
C27A—C28A	1.527 (2)	C29B—H29C	0.9900
C27A—H27A	0.9900	C29B—H29D	0.9900
C27A—H27B	0.9900	C30B—C31B	1.525 (2)
C28A—C29A	1.519 (2)	C30B—H30C	0.9900
C28A—H28A	0.9900	C30B—H30D	0.9900
C28A—H28B	0.9900	C31B—H31D	0.9800
C29A—C30A	1.528 (3)	C31B—H31E	0.9800
C29A—H29A	0.9900	C31B—H31F	0.9800
C29A—H29B	0.9900	C32B—H32D	0.9800
C30A—C31A	1.509 (3)	C32B—H32E	0.9800
C30A—H30A	0.9900	C32B—H32F	0.9800
C30A—H30B	0.9900		
			
C5A—S1A—C7A	93.53 (7)	H32A—C32A—H32B	109.5
C5B—S1B—C7B	93.16 (7)	C5A—C32A—H32C	109.5
F1B—C1A—F1A	107.96 (16)	H32A—C32A—H32C	109.5
F1B—C1A—C2A	112.29 (14)	H32B—C32A—H32C	109.5
F1A—C1A—C2A	109.58 (16)	F3B—C2B—F2B	106.49 (16)
F1B—C1A—C2B	112.38 (16)	F3B—C2B—C3B	113.45 (14)
F1A—C1A—C2B	109.83 (14)	F2B—C2B—C3B	110.76 (15)
C2A—C1A—C2B	104.78 (13)	F3B—C2B—C1A	110.70 (15)
F2A—C2A—F3A	105.95 (14)	F2B—C2B—C1A	109.43 (15)
F2A—C2A—C3A	112.71 (14)	C3B—C2B—C1A	106.02 (13)
F3A—C2A—C3A	111.58 (14)	C3A—C3B—C4B	128.12 (13)
F2A—C2A—C1A	111.51 (15)	C3A—C3B—C2B	111.42 (13)
F3A—C2A—C1A	109.31 (14)	C4B—C3B—C2B	120.40 (12)
C3A—C2A—C1A	105.84 (12)	C5B—C4B—C6B	112.97 (12)
C3B—C3A—C4A	129.04 (13)	C5B—C4B—C3B	123.82 (12)
C3B—C3A—C2A	111.08 (13)	C6B—C4B—C3B	123.21 (13)
C4A—C3A—C2A	119.87 (12)	C4B—C5B—C32B	129.58 (13)
C5A—C4A—C6A	112.67 (13)	C4B—C5B—S1B	110.46 (10)
C5A—C4A—C3A	125.03 (13)	C32B—C5B—S1B	119.94 (11)
C6A—C4A—C3A	122.30 (13)	C7B—C6B—C4B	113.19 (13)
C4A—C5A—C32A	130.37 (14)	C7B—C6B—H6B	123.4
C4A—C5A—S1A	110.23 (10)	C4B—C6B—H6B	123.4
C32A—C5A—S1A	119.35 (11)	C6B—C7B—C8B	128.28 (13)
C7A—C6A—C4A	113.68 (13)	C6B—C7B—S1B	110.20 (10)
C7A—C6A—H6A	123.2	C8B—C7B—S1B	121.51 (11)
C4A—C6A—H6A	123.2	C13B—C8B—C9B	118.34 (12)
C6A—C7A—C8A	127.79 (13)	C13B—C8B—C7B	120.01 (13)
C6A—C7A—S1A	109.87 (10)	C9B—C8B—C7B	121.63 (13)
C8A—C7A—S1A	122.17 (11)	C10B—C9B—C8B	120.40 (14)
C13A—C8A—C9A	117.77 (13)	C10B—C9B—H9B	119.8
C13A—C8A—C7A	121.78 (13)	C8B—C9B—H9B	119.8
C9A—C8A—C7A	120.30 (14)	F5B—C10B—C9B	115.8 (2)
C10A—C9A—C8A	120.85 (15)	F5B—C10B—C11B	121.7 (2)
C10A—C9A—H9A	119.6	C9B—C10B—C11B	122.41 (14)
C8A—C9A—H9A	119.6	F5B—C10B—H10B	3.9
C9A—C10A—C11A	122.98 (14)	C9B—C10B—H10B	118.8
C9A—C10A—F5A	112.0 (3)	C11B—C10B—H10B	118.8
C11A—C10A—F5A	121.9 (3)	C12B—C11B—C10B	115.45 (12)
C9A—C10A—H10A	118.5	C12B—C11B—C14B	122.25 (13)
C11A—C10A—H10A	118.5	C10B—C11B—C14B	122.19 (13)
F5A—C10A—H10A	19.0	F4B—C12B—C13B	117.17 (13)
C10A—C11A—C12A	114.57 (13)	F4B—C12B—C11B	118.96 (12)
C10A—C11A—C14A	120.29 (13)	C13B—C12B—C11B	123.77 (13)
C12A—C11A—C14A	125.12 (14)	F4B—C12B—H12B	3.5
F4A—C12A—C13A	115.65 (13)	C13B—C12B—H12B	118.1
F4A—C12A—C11A	120.24 (13)	C11B—C12B—H12B	118.1
C13A—C12A—C11A	124.05 (14)	C12B—C13B—C8B	119.62 (13)
F4A—C12A—H12A	3.4	C12B—C13B—H13B	120.2
C13A—C12A—H12A	118.0	C8B—C13B—H13B	120.2
C11A—C12A—H12A	118.0	C19B—C14B—C15B	118.22 (12)
C12A—C13A—C8A	119.74 (14)	C19B—C14B—C11B	121.43 (13)
C12A—C13A—H13A	120.1	C15B—C14B—C11B	120.32 (13)
C8A—C13A—H13A	120.1	C16B—C15B—C14B	120.78 (13)
C15A—C14A—C19A	117.19 (12)	C16B—C15B—H15B	119.6
C15A—C14A—C11A	119.32 (13)	C14B—C15B—H15B	119.6
C19A—C14A—C11A	123.46 (13)	C15B—C16B—C17B	120.35 (13)
C16A—C15A—C14A	122.32 (13)	C15B—C16B—H16B	119.8
C16A—C15A—H15A	118.8	C17B—C16B—H16B	119.8
C14A—C15A—H15A	118.8	O20B—C17B—C18B	125.06 (13)
C17A—C16A—C15A	119.33 (13)	O20B—C17B—C16B	115.21 (13)
C17A—C16A—H16A	120.3	C18B—C17B—C16B	119.73 (12)
C15A—C16A—H16A	120.3	C17B—C18B—C19B	119.25 (13)
O20A—C17A—C16A	124.03 (12)	C17B—C18B—H18B	120.4
O20A—C17A—C18A	116.43 (12)	C19B—C18B—H18B	120.4
C16A—C17A—C18A	119.54 (12)	C14B—C19B—C18B	121.65 (13)
C19A—C18A—C17A	120.54 (13)	C14B—C19B—H19B	119.2
C19A—C18A—H18A	119.7	C18B—C19B—H19B	119.2
C17A—C18A—H18A	119.7	C17B—O20B—C21B	118.34 (12)
C18A—C19A—C14A	120.98 (13)	O20B—C21B—C22B	106.99 (12)
C18A—C19A—H19A	119.5	O20B—C21B—H21C	110.3
C14A—C19A—H19A	119.5	C22B—C21B—H21C	110.3
C17A—O20A—C21A	116.96 (11)	O20B—C21B—H21D	110.3
O20A—C21A—C22A	107.35 (11)	C22B—C21B—H21D	110.3
O20A—C21A—H21A	110.2	H21C—C21B—H21D	108.6
C22A—C21A—H21A	110.2	C21B—C22B—C23B	113.15 (12)
O20A—C21A—H21B	110.2	C21B—C22B—H22C	108.9
C22A—C21A—H21B	110.2	C23B—C22B—H22C	108.9
H21A—C21A—H21B	108.5	C21B—C22B—H22D	108.9
C21A—C22A—C23A	113.06 (12)	C23B—C22B—H22D	108.9
C21A—C22A—H22A	109.0	H22C—C22B—H22D	107.8
C23A—C22A—H22A	109.0	C22B—C23B—C24B	112.01 (12)
C21A—C22A—H22B	109.0	C22B—C23B—H23C	109.2
C23A—C22A—H22B	109.0	C24B—C23B—H23C	109.2
H22A—C22A—H22B	107.8	C22B—C23B—H23D	109.2
C24A—C23A—C22A	111.57 (12)	C24B—C23B—H23D	109.2
C24A—C23A—H23A	109.3	H23C—C23B—H23D	107.9
C22A—C23A—H23A	109.3	C25B—C24B—C23B	113.76 (12)
C24A—C23A—H23B	109.3	C25B—C24B—H24C	108.8
C22A—C23A—H23B	109.3	C23B—C24B—H24C	108.8
H23A—C23A—H23B	108.0	C25B—C24B—H24D	108.8
C23A—C24A—C25A	114.52 (12)	C23B—C24B—H24D	108.8
C23A—C24A—H24A	108.6	H24C—C24B—H24D	107.7
C25A—C24A—H24A	108.6	C24B—C25B—C26B	112.95 (12)
C23A—C24A—H24B	108.6	C24B—C25B—H25C	109.0
C25A—C24A—H24B	108.6	C26B—C25B—H25C	109.0
H24A—C24A—H24B	107.6	C24B—C25B—H25D	109.0
C26A—C25A—C24A	111.95 (12)	C26B—C25B—H25D	109.0
C26A—C25A—H25A	109.2	H25C—C25B—H25D	107.8
C24A—C25A—H25A	109.2	C27B—C26B—C25B	113.48 (12)
C26A—C25A—H25B	109.2	C27B—C26B—H26C	108.9
C24A—C25A—H25B	109.2	C25B—C26B—H26C	108.9
H25A—C25A—H25B	107.9	C27B—C26B—H26D	108.9
C25A—C26A—C27A	114.25 (12)	C25B—C26B—H26D	108.9
C25A—C26A—H26A	108.7	H26C—C26B—H26D	107.7
C27A—C26A—H26A	108.7	C26B—C27B—C28B	113.71 (12)
C25A—C26A—H26B	108.7	C26B—C27B—H27C	108.8
C27A—C26A—H26B	108.7	C28B—C27B—H27C	108.8
H26A—C26A—H26B	107.6	C26B—C27B—H27D	108.8
C28A—C27A—C26A	112.96 (13)	C28B—C27B—H27D	108.8
C28A—C27A—H27A	109.0	H27C—C27B—H27D	107.7
C26A—C27A—H27A	109.0	C29B—C28B—C27B	112.63 (12)
C28A—C27A—H27B	109.0	C29B—C28B—H28C	109.1
C26A—C27A—H27B	109.0	C27B—C28B—H28C	109.1
H27A—C27A—H27B	107.8	C29B—C28B—H28D	109.1
C29A—C28A—C27A	114.62 (13)	C27B—C28B—H28D	109.1
C29A—C28A—H28A	108.6	H28C—C28B—H28D	107.8
C27A—C28A—H28A	108.6	C28B—C29B—C30B	113.73 (12)
C29A—C28A—H28B	108.6	C28B—C29B—H29C	108.8
C27A—C28A—H28B	108.6	C30B—C29B—H29C	108.8
H28A—C28A—H28B	107.6	C28B—C29B—H29D	108.8
C28A—C29A—C30A	113.43 (15)	C30B—C29B—H29D	108.8
C28A—C29A—H29A	108.9	H29C—C29B—H29D	107.7
C30A—C29A—H29A	108.9	C31B—C30B—C29B	112.79 (13)
C28A—C29A—H29B	108.9	C31B—C30B—H30C	109.0
C30A—C29A—H29B	108.9	C29B—C30B—H30C	109.0
H29A—C29A—H29B	107.7	C31B—C30B—H30D	109.0
C31A—C30A—C29A	114.55 (17)	C29B—C30B—H30D	109.0
C31A—C30A—H30A	108.6	H30C—C30B—H30D	107.8
C29A—C30A—H30A	108.6	C30B—C31B—H31D	109.5
C31A—C30A—H30B	108.6	C30B—C31B—H31E	109.5
C29A—C30A—H30B	108.6	H31D—C31B—H31E	109.5
H30A—C30A—H30B	107.6	C30B—C31B—H31F	109.5
C30A—C31A—H31A	109.5	H31D—C31B—H31F	109.5
C30A—C31A—H31B	109.5	H31E—C31B—H31F	109.5
H31A—C31A—H31B	109.5	C5B—C32B—H32D	109.5
C30A—C31A—H31C	109.5	C5B—C32B—H32E	109.5
H31A—C31A—H31C	109.5	H32D—C32B—H32E	109.5
H31B—C31A—H31C	109.5	C5B—C32B—H32F	109.5
C5A—C32A—H32A	109.5	H32D—C32B—H32F	109.5
C5A—C32A—H32B	109.5	H32E—C32B—H32F	109.5
			
F1B—C1A—C2A—F2A	−105.63 (18)	C2A—C1A—C2B—F3B	−130.47 (16)
F1A—C1A—C2A—F2A	14.34 (19)	F1B—C1A—C2B—F2B	−9.7 (2)
C2B—C1A—C2A—F2A	132.12 (15)	F1A—C1A—C2B—F2B	−129.92 (18)
F1B—C1A—C2A—F3A	11.2 (2)	C2A—C1A—C2B—F2B	112.46 (17)
F1A—C1A—C2A—F3A	131.15 (15)	F1B—C1A—C2B—C3B	−129.22 (16)
C2B—C1A—C2A—F3A	−111.07 (15)	F1A—C1A—C2B—C3B	110.59 (17)
F1B—C1A—C2A—C3A	131.47 (16)	C2A—C1A—C2B—C3B	−7.03 (18)
F1A—C1A—C2A—C3A	−108.56 (15)	C4A—C3A—C3B—C4B	5.9 (3)
C2B—C1A—C2A—C3A	9.22 (18)	C2A—C3A—C3B—C4B	−172.90 (14)
F2A—C2A—C3A—C3B	−130.70 (15)	C4A—C3A—C3B—C2B	−177.08 (15)
F3A—C2A—C3A—C3B	110.24 (15)	C2A—C3A—C3B—C2B	4.12 (19)
C1A—C2A—C3A—C3B	−8.56 (18)	F3B—C2B—C3B—C3A	123.74 (16)
F2A—C2A—C3A—C4A	50.4 (2)	F2B—C2B—C3B—C3A	−116.56 (16)
F3A—C2A—C3A—C4A	−68.68 (18)	C1A—C2B—C3B—C3A	2.05 (19)
C1A—C2A—C3A—C4A	172.51 (14)	F3B—C2B—C3B—C4B	−59.0 (2)
C3B—C3A—C4A—C5A	43.2 (2)	F2B—C2B—C3B—C4B	60.7 (2)
C2A—C3A—C4A—C5A	−138.06 (16)	C1A—C2B—C3B—C4B	179.33 (14)
C3B—C3A—C4A—C6A	−137.44 (16)	C3A—C3B—C4B—C5B	47.9 (2)
C2A—C3A—C4A—C6A	41.3 (2)	C2B—C3B—C4B—C5B	−128.86 (16)
C6A—C4A—C5A—C32A	−176.13 (15)	C3A—C3B—C4B—C6B	−131.24 (17)
C3A—C4A—C5A—C32A	3.3 (3)	C2B—C3B—C4B—C6B	52.0 (2)
C6A—C4A—C5A—S1A	1.47 (16)	C6B—C4B—C5B—C32B	−176.92 (14)
C3A—C4A—C5A—S1A	−179.14 (12)	C3B—C4B—C5B—C32B	3.8 (2)
C7A—S1A—C5A—C4A	−1.16 (12)	C6B—C4B—C5B—S1B	1.18 (16)
C7A—S1A—C5A—C32A	176.75 (13)	C3B—C4B—C5B—S1B	−178.06 (11)
C5A—C4A—C6A—C7A	−1.12 (19)	C7B—S1B—C5B—C4B	−0.29 (12)
C3A—C4A—C6A—C7A	179.48 (13)	C7B—S1B—C5B—C32B	178.02 (12)
C4A—C6A—C7A—C8A	175.55 (14)	C5B—C4B—C6B—C7B	−1.77 (18)
C4A—C6A—C7A—S1A	0.22 (16)	C3B—C4B—C6B—C7B	177.48 (13)
C5A—S1A—C7A—C6A	0.53 (12)	C4B—C6B—C7B—C8B	−177.73 (14)
C5A—S1A—C7A—C8A	−175.10 (12)	C4B—C6B—C7B—S1B	1.49 (16)
C6A—C7A—C8A—C13A	−169.33 (15)	C5B—S1B—C7B—C6B	−0.69 (12)
S1A—C7A—C8A—C13A	5.5 (2)	C5B—S1B—C7B—C8B	178.59 (12)
C6A—C7A—C8A—C9A	6.2 (2)	C6B—C7B—C8B—C13B	−22.3 (2)
S1A—C7A—C8A—C9A	−178.99 (12)	S1B—C7B—C8B—C13B	158.51 (11)
C13A—C8A—C9A—C10A	0.0 (2)	C6B—C7B—C8B—C9B	156.00 (16)
C7A—C8A—C9A—C10A	−175.66 (15)	S1B—C7B—C8B—C9B	−23.1 (2)
C8A—C9A—C10A—C11A	−1.2 (3)	C13B—C8B—C9B—C10B	1.2 (2)
C8A—C9A—C10A—F5A	159.1 (3)	C7B—C8B—C9B—C10B	−177.13 (14)
C9A—C10A—C11A—C12A	2.0 (2)	C8B—C9B—C10B—F5B	177.0 (3)
F5A—C10A—C11A—C12A	−156.4 (3)	C8B—C9B—C10B—C11B	−0.2 (2)
C9A—C10A—C11A—C14A	−178.99 (15)	F5B—C10B—C11B—C12B	−178.1 (3)
F5A—C10A—C11A—C14A	22.7 (4)	C9B—C10B—C11B—C12B	−1.0 (2)
C10A—C11A—C12A—F4A	175.25 (14)	F5B—C10B—C11B—C14B	−1.9 (4)
C14A—C11A—C12A—F4A	−3.7 (2)	C9B—C10B—C11B—C14B	175.18 (14)
C10A—C11A—C12A—C13A	−1.9 (2)	C10B—C11B—C12B—F4B	177.47 (13)
C14A—C11A—C12A—C13A	179.16 (14)	C14B—C11B—C12B—F4B	1.3 (2)
F4A—C12A—C13A—C8A	−176.34 (14)	C10B—C11B—C12B—C13B	1.3 (2)
C11A—C12A—C13A—C8A	0.9 (2)	C14B—C11B—C12B—C13B	−174.90 (13)
C9A—C8A—C13A—C12A	0.1 (2)	F4B—C12B—C13B—C8B	−176.55 (13)
C7A—C8A—C13A—C12A	175.73 (14)	C11B—C12B—C13B—C8B	−0.3 (2)
C10A—C11A—C14A—C15A	26.1 (2)	C9B—C8B—C13B—C12B	−1.0 (2)
C12A—C11A—C14A—C15A	−154.98 (15)	C7B—C8B—C13B—C12B	177.41 (13)
C10A—C11A—C14A—C19A	−151.76 (15)	C12B—C11B—C14B—C19B	−46.0 (2)
C12A—C11A—C14A—C19A	27.2 (2)	C10B—C11B—C14B—C19B	138.05 (15)
C19A—C14A—C15A—C16A	3.3 (2)	C12B—C11B—C14B—C15B	131.95 (15)
C11A—C14A—C15A—C16A	−174.65 (13)	C10B—C11B—C14B—C15B	−44.0 (2)
C14A—C15A—C16A—C17A	−1.3 (2)	C19B—C14B—C15B—C16B	1.1 (2)
C15A—C16A—C17A—O20A	179.20 (13)	C11B—C14B—C15B—C16B	−176.87 (13)
C15A—C16A—C17A—C18A	−1.7 (2)	C14B—C15B—C16B—C17B	−1.1 (2)
O20A—C17A—C18A—C19A	−178.38 (12)	C15B—C16B—C17B—O20B	179.34 (13)
C16A—C17A—C18A—C19A	2.4 (2)	C15B—C16B—C17B—C18B	−0.3 (2)
C17A—C18A—C19A—C14A	−0.3 (2)	O20B—C17B—C18B—C19B	−178.00 (13)
C15A—C14A—C19A—C18A	−2.5 (2)	C16B—C17B—C18B—C19B	1.6 (2)
C11A—C14A—C19A—C18A	175.35 (13)	C15B—C14B—C19B—C18B	0.2 (2)
C16A—C17A—O20A—C21A	−6.48 (19)	C11B—C14B—C19B—C18B	178.19 (13)
C18A—C17A—O20A—C21A	174.35 (12)	C17B—C18B—C19B—C14B	−1.6 (2)
C17A—O20A—C21A—C22A	−176.30 (11)	C18B—C17B—O20B—C21B	−1.6 (2)
O20A—C21A—C22A—C23A	179.82 (11)	C16B—C17B—O20B—C21B	178.79 (12)
C21A—C22A—C23A—C24A	178.18 (12)	C17B—O20B—C21B—C22B	171.24 (12)
C22A—C23A—C24A—C25A	179.34 (12)	O20B—C21B—C22B—C23B	175.02 (11)
C23A—C24A—C25A—C26A	−178.77 (12)	C21B—C22B—C23B—C24B	179.14 (12)
C24A—C25A—C26A—C27A	−178.05 (13)	C22B—C23B—C24B—C25B	179.04 (12)
C25A—C26A—C27A—C28A	175.55 (14)	C23B—C24B—C25B—C26B	−178.33 (12)
C26A—C27A—C28A—C29A	−163.14 (16)	C24B—C25B—C26B—C27B	177.23 (12)
C27A—C28A—C29A—C30A	179.31 (17)	C25B—C26B—C27B—C28B	179.52 (12)
C28A—C29A—C30A—C31A	−173.09 (18)	C26B—C27B—C28B—C29B	−177.11 (12)
F1B—C1A—C2B—F3B	107.34 (18)	C27B—C28B—C29B—C30B	−176.58 (12)
F1A—C1A—C2B—F3B	−12.9 (2)	C28B—C29B—C30B—C31B	−177.79 (13)
